# Proteomic characterization of *Naja mandalayensis* venom

**DOI:** 10.1590/1678-9199-JVATITD-2020-0125

**Published:** 2021-07-30

**Authors:** Emídio Beraldo, Guilherme Rabelo Coelho, Juliana Mozer Sciani, Daniel Carvalho Pimenta

**Affiliations:** 1 Laboratory of Biochemistry and Biophysics, Butantan Institute, São Paulo, SP, Brazil.; 2 Graduation Program in Science - Toxinology, Butantan Institute, São Paulo, SP, Brazil.; 3 Multidisciplinary Research Laboratory, São Francisco University, Bragança Paulista, SP, Brazil.

**Keywords:** Naja mandalayensis, Proteome, Spitting cobra, Three-finger toxins, SVMP, Enzymes

## Abstract

**Background:**

*Naja mandalayensis* is a spitting cobra from Myanmar. To the best of our knowledge, no studies on this venom composition have been conducted so far. On the other hand, few envenomation descriptions state that it elicits mainly local inflammation in the victims’ eyes, the preferred target of this spiting cobra. Symptoms would typically include burning and painful sensation, conjunctivitis, edema and temporary loss of vision.

**Methods:**

We have performed a liquid-chromatography (C18-RP-HPLC) mass spectrometry (ESI-IT-TOF/MS) based approach in order to biochemically characterize *N. mandalayensis* venom.

**Results:**

A wide variety of three-finger toxins (cardiotoxins) and metallopeptidases were detected. Less abundant, but still representative, were cysteine-rich secretory proteins, L-amino-acid oxidases, phospholipases A_2_, venom 5'-nucleotidase and a serine peptidase inhibitor. Other proteins were present, but were detected in a relatively small concentration.

**Conclusion:**

The present study set the basis for a better comprehension of the envenomation from a molecular perspective and, by increasing the interest and information available for this species, allows future venom comparisons among cobras and their diverse venom proteins.

## Background

*Naja mandalayensis* is a spitting cobra described for the first time in 2000 by Slowinski and Wüster [1], which is endemic in the central region of Myanmar, covering the Mandalay, Magwe and Sagaing regions. Its distribution corresponds to the dry zone of Myanmar, originally an Acacia savanna that is currently an agricultural region. However, this species has adapted and thrived in the agricultural fields and village surroundings. *Naja mandalayensis* belongs to a parafiletic group that comprises *N. mossambica*, *N. annulifera* and it is closer to *N. siamensis*, *N. kaouthia* and *N. atra*. [1].

The first description of a *N. mandalayensis* envenoming was published by a Myanmar research group that reported eight patients that were spat in the eyes by this venomous snake. Patients reported a burning and painful sensation (ophthalmia) and presented conjunctivitis, edema and temporary loss of vision [2, 3]. This is very similar to the *N. mossambica* accident, whose venom, when in contact with eyes, induces lacrimation, blepharospasm, conjunctivitis, keratitis and iritis miosis or mydriasis, symptoms related to inflammatory and cytotoxic compounds [4]. Slowinski, in the year 2000, published a letter depicting a self-accident with a *N. mandalayensis* specimen that spat venom directly to the author’s eyes inducing a burning painful reaction, and conjunctivitis, according to the report [1].

Despite the lack of reports on *N. mandalayensis* accidents, they remain a serious public health issue in Myanmar, as the snakebite incidence is high, and 70% of the population live in rural areas. Treatment for these patients is mainly based on antivenom administration (although traditional methods, such as herbal medicines, electric shock and suction are still being used). Regardless of the availability of antivenoms, Myanmar faces difficulties with the supply of antivenom, particularly in the rural areas [5]. Recently, it has been reported that antiserum used in the region of Myanmar displays lower efficacy against *N. mandalayensis* [3].

Considering that snakebite is a serious medical problem in rural areas of Myanmar and the data available on *N. mandalayensis* venom and envenomation is still scarce, and taking into account the relevance of understanding the biochemical composition of its venom, we have biochemically described, for the first time, the protein composition of its venom by using high performance liquid chromatography and mass spectrometry ESI-IT-TOF (proteomic) techniques.

## Methods

### Reagents

All reagents used in the present study are of analytical grade and obtained from Merck (Merck KGaA, Darmstadt, Germany and/or its affiliates), unless otherwise stated.

### Venom attainment

A stablished partnership between Butantan Institute and the Ministry of Health of Myanmar, encompassed in a project called ‘Methods and techniques improvement for antivenom production in Myanmar’ granted us access to the pooled venom sample - from captivity individuals. 

### Sample preparation

The first step was to decomplex the venom in order to improve mass spectrometric analyses. Briefly, 10 mg lyophilized *N. mandalayenses* venom were resuspended in 0.1% Trifluoroacetic Acid (TFA) and centrifuged (10,000 x *g*) for 10 minutes, at 4ºC. The supernatant was then analyzed and fractionated by Reversed-Phase High Performance Liquid Chromatography (RP-HPLC) in a Shimadzu Prominence binary system (Shimadzu, Kyoto, Japan), coupled to a C18 analytical column (Supelco, 250 x 4.6 mm, 10 µm). UV detection was performed (SPDM 20A, Shimadzu, λ = 214 nm) and separation was achieved by a linear gradient of 0-40% solvent B (90% acetonitrile, containing 0.1% TFA) over A (0.1% TFA) for 40 minutes at a constant flow of 1 mL.min^-1^.

### Mass spectrometry analyses

Manually collected fractions (50 µL aliquots) were submitted to in-solution digestion under the following conditions: (1) 5 µL DTT (100 mM dithiothreitol) were added for 30 minutes at 60ºC; (2) 2.5 µL of iodoacetamide (200 mM) added for another 30 minutes at room temperature and protected from light; (3) sample incubation for at least 12 hours at room temperature with 10 µL of trypsin (40 ng/µL in 100 mM ammonium bicarbonate). The reaction was stopped by adding 50% ACN/5% TFA.

The samples then were analyzed by liquid chromatography-mass spectrometry in an ESI-IT-TOF instrument coupled to a UPLC 20A Prominence (Shimadzu, Kyoto, Japan). Samples (15 µL aliquots) were loaded into a C18 column (Kinetex C18, 5 μm; 50 × 2.1 mm) and fractionated by a binary gradient employing as solvents (A) water: DMSO: acid (949: 50: 1) and (B) ACN: DMSO: water: acid (850: 50: 99: 1). An elution gradient of 0-40% B was applied for 35 minutes at a constant flow of 0.2 mL.min^-1^ after initial isocratic elution for 5 minutes. The eluates were monitored by a Shimadzu SPD-M20A PDA detector before being injected into the mass spectrometer.

The interface was kept at 4.5 kV and 275ºC. Detector operated at 1.95 kV and the argon collision induced fragmentation was set at 55 ‘energy’ value. MS spectra were acquired in positive mode, in the 350-1400 m/z range and MS/MS spectra were collected in the 50 to 1950 m/z range.

### Proteomic data processing

Raw LCD LCMSolution Shimadzu data were converted into MGF by the LCMSolution (PRIDE) tool and then loaded into Peaks Studio V7.0 (BSI, Canada). Data were processed according to the following parameters: MS and MS/MS error mass were 0.1 Da; methionine oxidation and carbamidomethylation as variable and fixed modification, respectively; trypsin as cleaving enzyme; maximum missed cleavages (3), maximum variable PTMs per peptide (3) and non-specific cleavage (both); the false discovery rate was adjusted to ≤ 0.5%; only proteins with score ≥20 and containing at least 1 unique peptide were considered in this study. Data were analyzed against a *Naja* protein database (963 entries) compiled on January 2020 and built by retrieving all UniProt entries associated with this taxon, although broad searches against the whole UniProt were performed, as well, as quality controls (data not shown).

## Results and discussion

**Figure 1.**
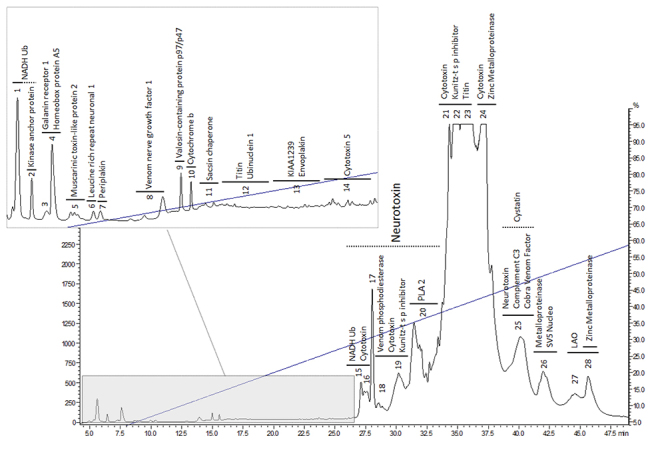
C18-RP-HPLC representative profile of *N. mandalayensis* venom solution containing the fraction numbering as well as the toxin identification attained after the fraction proteomic processing.

Sample preparation is a critical step, required prior to any analytical step, particularly when the relative concentration range of the sample components is wide. One possible approach is to decomplex the sample by RP-HPLC [6]. In this work, we have chosen this approach in order to make it possible to identify both major toxins and minor components. Figure 1 presents the annotated RP-HPLC chromatogram, in which the proteomically identified proteins are indicated above the correspondent chromatographic fraction. It is possible to observe that the dynamic concentration range of toxins, in this particular venom, is very wide. For example, RT~35´ contains six abundant proteins, which detection saturated the UV signal, whereas RT ~5-25 contains sixteen minor proteins, which UV detection level was, at least, 15 times less intense than the largest signal. Thus, if shotgun proteomic strategy would be performed, less intense proteins would not likely be detected, as presented here. Although other sample preparation methods are available, such as batch-ion exchange chromatography [7], we have chosen to perform RP-HPLC based separation for our previous studies have demonstrated the non-viscous venoms can be efficiently fractionated by this technique [8, 9].

Therefore, this decomplexation step - which was not intended to yield an ‘analytical’ profile, with the best possible resolution and signal to noise ratio - made it possible to normalize the protein contents in each individual fraction, leading to an optimal sample processing (reduction, alkylation and trypsinization) and consequent enhanced mass spectrometric detection of the normalized tryptic peptides.

**Figure 2.**
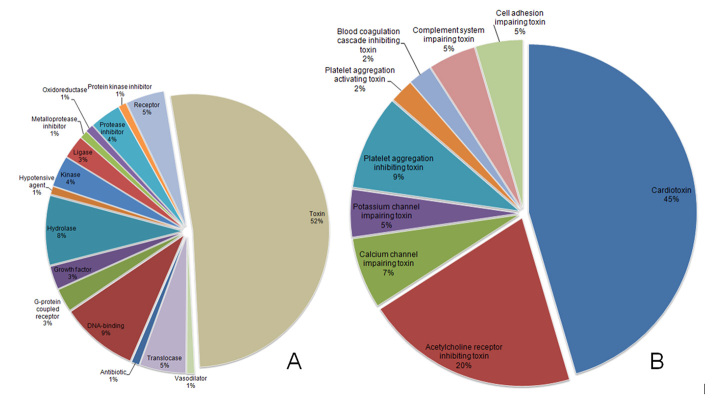
(A) Relative protein distribution (according to their UniProt ‘molecular function’ identifier) of the proteomically identified proteins in *N. mandalayensis* venom. (B) Relative toxin (from ‘A’) distribution (according to their UniProt ‘mechanism of action’ identifier) of this proteomic subset.

Following sample preparation and proteomic processing, *N. mandalayeneses* venom revealed itself to be a complex mixture of proteins (Figure 2). The UniProt GO molecular function annotation analyses led to the protein distribution presented in Figure 2A. Not surprising, more than half of the identified proteins fell into the ‘toxin’ category. In order to better understand these toxins, a second pie-chart graph was assembled analyzing only the ‘toxin’ keyword matched proteins (Figure 2B). This toxin distribution is in agreement with the recent work of Kazandjian et al. [10] that have elegantly studied the convergent evolution of the venom components of spitting cobras.

Among the identified proteins, some known venom toxins could be detected. Namely: venom zinc metalloproteinase (SVMP) (Table 1), phospholipase A_2_ (PL A_2_), L-amino acid oxidase (LAAO), cysteine-rich venom protein (CRISP), venom 5-nucleotidase (V5N) and venom nerve growth factor (VNGF) (Table 2) [11, 12]. Moreover, other proteins, such as venom phosphodiesterase and NADH-related enzymes could be identified, also shown in Table 2. The complete list of the ‘other’ identified proteins is presented in the Additional file 1. 

**Table 1.** Known metallopeptidases matched to the proteomically identified toxins from *N. mandalayensis* venom.

Description	Accession	-10lgP^1^	Peptides^2^	Avg. mass^3^ (Da)	Organism^4^
Zinc metallopeptidase-disintegrin-like atragin	D3TTC2	319.7	25	69181	*N. atra*
Zinc metallopeptidase-disintegrin-like cobrin	Q9PVK7	259.8	23	67662	*N. kaouthia*
Hemorrhagic metallopeptidase-disintegrin-like kaouthiagin	P82942	256.8	16	44493	
Snake venom metallopeptidase-disintegrin-like mocarhagin	Q10749	249.9	20	68176	*N. mossambica*
Zinc metallopeptidase-disintegrin-like kaouthiagin-like	D3TTC1	237.4	22	66292	*N. atra*
Zinc metallopeptidase-disintegrin-like atrase-A	D5LMJ3	214.4	23	68254	

^1^Peaks Suite confidence parameter. The cutoff was set > 50. ^2^Matched peptides supporting the protein identification. ^3^Retrieved theoretical value. ^4^Species from which the matching toxins were identified.

**Table 2.** Other known enzymes matched to the proteomically identified toxins from *N. mandalayensis* venom.

Description	Accession	-10lgP^1^	Peptides^2^	Avg. mass^3^ (Da)	Organism^4^
L-amino-acid oxidase	A8QL58	323.01	43	57963	*N. atra*
Snake venom 5'-nucleotidase (Fragment)	A0A2I4HXH5	216.74	25	58198	
Venom phosphodiesterase	A0A2D0TC04	186.34	22	94616	
Basic phospholipase A_2_ nigexine	P14556	56.76	2	13340	*N. pallida*
A kinase anchor protein 9	K4GX13	47.94	4	47420	*N. kaouthia*
NADH-ubiquinone oxidoreductase chain 2	A9X4E0	42.95	2	38341	*N. naja*
	Q2V505		2	38341	*N. atra*
	A0A4P2VGL4		2	37925	*N. kaouthia*
	B6DCF5		2	38321	*N. atra*
	B6DA70		2	38253	
	Q8W9X2	37.31	2	38059	*N. nivea*
NADH-ubiquinone oxidoreductase chain 4	D9YM78	35.69	2	23869	*N. arabica*
Phosphatidylinositol-4,5-bisphosphate 3-kinase catalytic subunit gamma	A0A5B9CKM0	30.63	2	37581	*N. atra*
	A0A5B9CL15		2	37581	*N. kaouthia*
	A0A5B9CMR9		2	37597	*N. atra*
NADH-ubiquinone oxidoreductase chain 4	A0A1W5PVT2	26.51	1	24190	*N. melanoleuca*
	A0A3G2KUZ9		1	24434	
	A0A3G2KV06		1	24390	
	A0A3G2KV13		1	24450	
	A0A3G2KV16		1	24420	
	A0A3G2KV47		1	24445	*N. peroescobari*
NADH dehydrogenase subunit 4	A0A3G2KUY9		1	24469	*N. guineensis*
	A0A3G2KV04		1	24481	
	A0A3G2KV05		1	24481	

^1^Peaks Suite confidence parameter. The cutoff was set > 25. ^2^Matched peptides supporting the protein identification. ^3^Retrieved theoretical value. ^4^Species from which the matching toxins were identified.

The pharmacological effect of the SVMPs in cobras has not been fully understood yet [13]. Few cobra SVMPs were biologically studied. Kaouthiagin (uniprot entry P82942) is an example: it seems to specifically bind to and cleave von Willebrand factor. In this sense, those authors speculate that this enzyme could be used as a pharmacological tool for functional studies of this factor [14].

**Figure 3.**
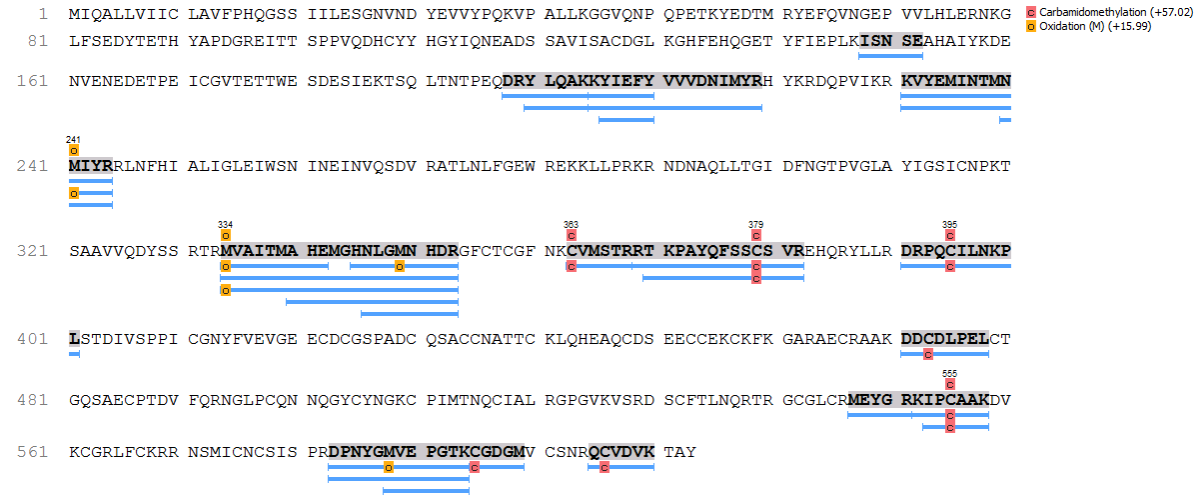
Coverage map representing the proteomic identification of SVMP (D3TTC2), according to Peaks Studio analyses. Blue bars represent the proteomically matched peptides, from *N. mandalayensis* venom, over the deposited sequence from *N. atra*. Letters ‘c’ and ‘o’ are the considered post-translation modifications selected for this analysis.

Mocarhagin (Q10749) is another *Naja* SVMP that alters the clotting homeostasis [15]. But, in general, most cobra’s SVMPs have only been associated with platelet aggregation inhibition [16]. In the present work we have observed that - for *N. mandalayensis* - SVMPs were the most abundant (Figure 1) proteins identified among the hydrolases. The proteomic analyses (Table 1) show that these enzymes matched SVMPs from three different *Naja* species. It is interesting to mention that the high peptide count presented in Table 1 not only indicates that several peptides were detected in the MS analyses (protein quantity), but also that these peptides are fairly distributed throughout the protein (rather than matching only the active site, for example), indicating that there is some degree of protein homology among these species (Figure 3) [17, 18, 19].

LAAO and PLA_2_ are commonly highlighted as enzymes of medical importance [20, 21] due to their abundance in the venom. These proteins are presented in Table 2, among others. The currently idenfied *Naja* LAAO (A8QL58) has little reported information regarding its pharmacological effects. Thus, this protein could present itself as an interesting molecule for further studies, for it is a remnant isoform from a diverse family of toxins. The here reported PLA_2_ (P14556), is similar to Nigexine - a basic phospholipase A_2_ from the venom of the spitting cobra *Naja nigricollis* - and may display an anticoagulant activity and affect the neuromuscular transmission [21, 22]. PLA_2_s from *N. naja* are considered lethal due to their neurotoxic activity, as previously observed in an experimental envenomation study [23]. Moreover, LAAOs, PLA_2_s and SVMPs have been associated to tissue inflammation and local necrosis of Elapidae snakes [24].

Also presented in Table 2 is the identification of Venom Phosphodiesterase (A0A2D0TC04) and V5N (A0A2I4HXH5), proteins that affect homeostasis and inhibit platelet aggregation induced by ADP, a consequence of both enzymes being able to degrade nucleotides [25-29]. According to Mitra and Bhattacharyya [30] who have isolated a phosphodiesterase from *Daboia russelli* venom with ADP hydrolytic activity, the platelet aggregation inhibition (in a human platelet rich plasma model) would be a consequence of the enzyme’s activity.

Some toxins, on the other hand, do not display a defined role in the venom yet, such as Phosphatidylinositol-4,5-bisphosphate 3-kinase (A0A5B9CKM0), the NADH metabolism-related enzymes (Table 2) and A-kinase anchor protein 9 (K4GX13). Although the latter is directly related to the control of the production of reactive oxygen species in cardiac stress, responsible for cardiomyocyte dysfunction and death [31].

Nonetheless, the majority of the identified proteins in *N. mandalayensis* venom were three-finger toxins (3FTx, Figure 2B), particularly cytotoxins (Table 3) and neurotoxins (Table 4). Such abundance of 3FTx has been reported by other authors for related species [10]. In a work that describes the proteome of *N. kaouthia* and *N. naja* venom, 3FTx were the most representative toxins found in an Elapidae database [32].

**Table 3.** Known 3FTx cytotoxins matched to the proteomically identified toxins from *N. mandalayensis* venom.

Description	Accession	-10lgP^1^	Peptides^2^	Avg. mass^3^ (Da)	Organism^4^
Cytotoxin 1	P01455	74.98	3	6696	*N. annulifera*
Cytotoxin 1d/1e	Q98958	129.4	9	8992	*N. atra*
Cytotoxin 2	P01442	82.64	4	9041	
	P01445	150.2	5	6745	*N. kaouthia*
	P01474	74.49	3	6850	*N. melanoleuca*
Cytotoxin 3	P01446	150.2	5	6717	*N. kaouthia*
Cytotoxin 4	P01452	90.91	3	6715	*N. mossambica*
	P01443	82.64	4	9084	*N. atra*
Cytotoxin 4N	Q9W6W9	103.9	5	9099	
Cytotoxin 5	P07525	139.9	4	6810	
	Q98961	128.6	4	9086	
	P24779	132.5	6	6654	*N. kaouthia*
Cytotoxin 5a	O73857	82.64	4	9041	*N. sputatrix*
Cytotoxin 5b	P60310		4	9055	
Cytotoxin 6	P80245	136.3	8	8980	*N. atra*
Cytotoxin 7	P86382	118.6	6	6792	*N. naja*
	P49122	54.72	4	9086	*N. atra*
Cytotoxin 8	Q91124	114.4	6	8900	
	P86540	98	5	6793	*N. naja*
Cytotoxin 10	P86541	78.19	3	6764	
Cytotoxin 11	P62394	50.31	2	6842	*N. haje haje*
	P62390		2	6842	*N. annulifera*
Cytotoxin 13	A0A0U4N5W4	150.2	5	7947	*N. naja*
Cytotoxin SP15d	P60309	152.2	9	6652	*N. atra*
Cytotoxin 16	A0A0U4W6K7	132.1	7	7967	*N. naja*
Cytotoxin homolog	P14541	83.26	2	6994	*N. kaouthia*
Three-finger toxin	E2ITZ4	124.5	4	9139	*N. atra*
	E2ITZ6	90.36	5	9858	
	E2ITZ7	76.54	3	8834	

^1^Peaks Suite confidence parameter. The cutoff was set > 50. ^2^Matched peptides supporting the protein identification. ^3^Retrieved theoretical value. ^4^Species from which the matching toxins were identified.

**Table 4.** Known neurotoxins from 3FTx and CRISP families matched to the proteomically identified toxins from *N. mandalayensis* venom.

Description	Accession	-10lgP^1^	Peptides^2^	Avg. mass^3^ (Da)	Organism^4^
Cysteine-rich venom protein kaouthin-1	P84805	185.4	9	26846	*N. kaouthia*
Cysteine-rich venom protein natrin-1	Q7T1K6		9	26882	*N. atra*
Cobrotoxin-b	P80958	124.5	4	9139	
Cobrotoxin-c	P59276	136.8	4	6859	*N. kaouthia*
Short neurotoxin 1	P60774		4	6818	*N. samarensis*
	P60773		4	6873	*N. philippinensis*
Neurotoxin 5	P60772		4	6818	*N. sputatrix*
Weak neurotoxin NNAM2	Q9YGI4	59.92	3	9899	*N. atra*
Weak neurotoxin 5	O42255		3	9806	*N. sputatrix*
Weak neurotoxin 6	O42256		3	9807	
Weak neurotoxin 8	Q802B3		3	9809	
Weak neurotoxin 9	Q9W7I3		3	9836	
Muscarinic toxin-like protein 2	P82463	46.55	3	7298	*N. kaouthia*

^1^Peaks Suite confidence parameter. The cutoff was set > 40. ^2^Matched peptides supporting the protein identification. ^3^Retrieved theoretical value. ^4^Species from which the matching toxins were identified.

In the present report, we could identify 19 different cytotoxins (Table 3) corresponding to approximately 45% (Figure 2B) of the toxins found. These molecules are also the ones displaying the largest presence of isoforms [33]. Their biological effects include systolic cardiac arrest and severe tissue necrosis, amongst others [34]. Moreover, most of these toxins are cytolytic to different cell lines, through a pore-forming activity on membranes. Nevertheless, their actual mechanism of action has not yet been completely elucidated [35].

The 3FTx neurotoxins (12 identifications, Table 4), accounted for about 32% of the toxins in the *N. mandalayensis* venom (Figure 2B). Elapid snake toxins are generally known to act on the nervous system, inhibiting the synaptic transmission by specifically and potently blocking a variety of ion channels, like Ca^2+^ and K^+^, as well as the nicotinic acetylcholine receptors [36, 37]. There are described CRISP toxins that also can behave as neurotoxins [38] by inhibiting Ca^2+^-activated K^+^ channels and voltage-gated K^+^ channels. Table 3 lists the 3FTx-neurotoxins found in the current work.

**Figure 4.**
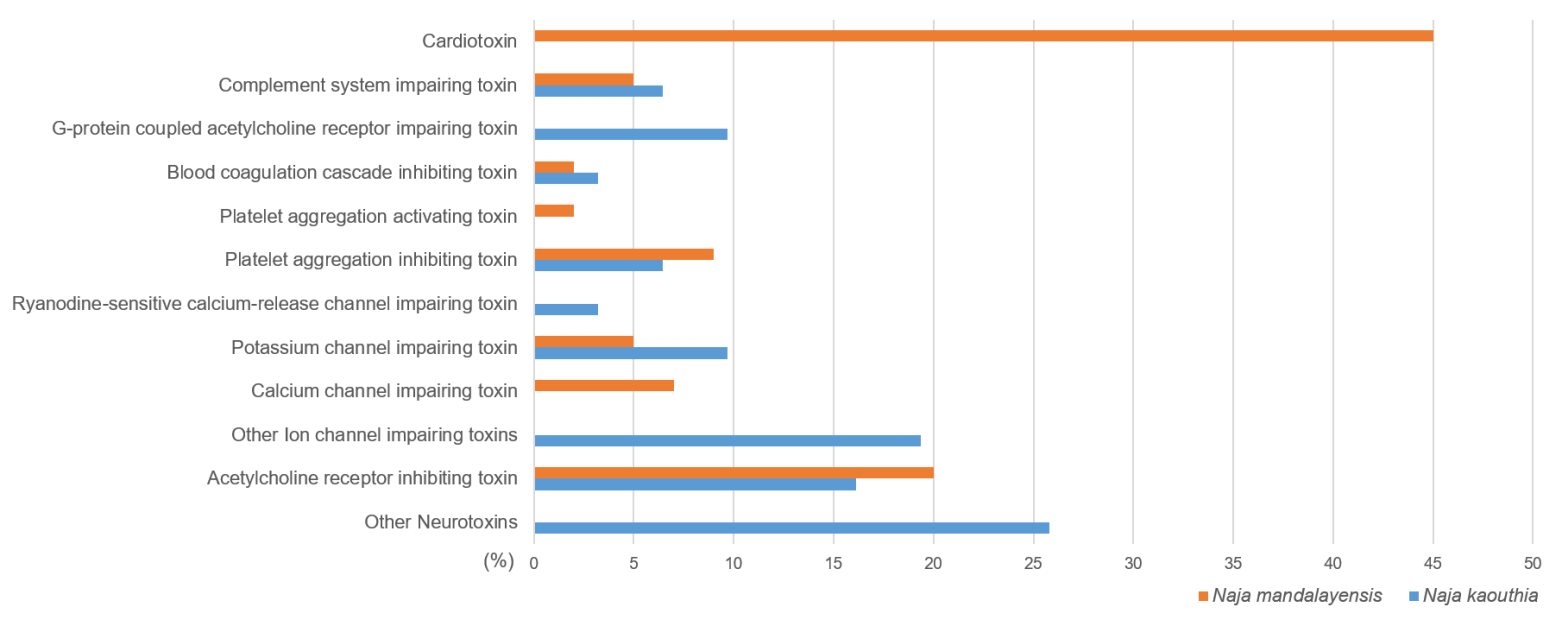
Relative toxin concentration distribution (%) for *N. mandalayensis* (current work) and *N. kaouthia* (UniProt data) venoms. Classification terms based on the UniProt ‘mechanism of action’.

In order to compare the present identified proteins groups/families and glimpse on their biological interest/relevance, we have compared the relative venom protein composition of *N. mandalayensis* venom with *N. kaouthia*, the closest phylogenetically relative according to Slowinski and Wüster [1]. This analysis is presented in Figure 4 and shows that homologous proteins display similar relative concentrations, such as acetylcholine receptor inhibitors, calcium channel inhibitors, platelet aggregation inhibitors and complement system inhibitors.

There were, however, *N. kaouthia* proteins that did not match any protein in the *N. mandalayensis* venom, such as ryanodine-sensitive calcium-release channel impairing and G-protein coupled acetylcholine receptor impairing. We cannot, though, relate the absence of these proteins - in the current study - to any actual lack of these proteins in *N. mandalayensis* venom. This event might be related to methodological/artefactual/biological phenomena that still need to be further explored. 

Noteworthy to mention is the high relative concentration of the cardiotoxins (3FTx) standing out for *N. mandalayensis* venom in comparison to *N. kaouthia*. A similar protein profile, however, has already been described for *Ophiophagus hannah* [25], a Malaysian king cobra. The transcriptome and proteome analyses revealed that 3FTx were the major venom component. *O. hannah*’s venom induces neurotoxicity and paralysis of the respiratory muscles and frequently provokes an extensive tissue necrosis and inflammation [25], similar to *N. mandalayensis* venom [2, 3]. 

Elapidae and Viperidae venoms have already been described to contain multiple peptidase inhibitors, most of them belonging to the Kunitz-type pancreatic trypsin inhibitors family [39,32]. Elapidae venoms, in particular, display strong inhibitory activity over mammalian serine peptidases, including trypsin, α-chymotrypsin, plasmin and kallikrein [40].

As presented in Figure 2A, peptidase inhibitors were also detected in the studied venom; however, at low levels (~1%). Nevertheless, due to the possible physo(patho)logical effects of peptidase inhibitors, we chose to present our findings regarding these molecules in Table 5. Two Kunitz-type serine protease inhibitor were detected and, according to Peaks Studio analyses, are identical (100% coverage, data not shown) to already sequence inhibitors from other *Najas* (P20229; P00986). Moreover, one cystatin already described for *N. atra*, (P81714), capable of inhibiting various cysteine proteases including cathepsin L, S, B and papain, were, also present in the venom[39]. However, in previous studies no toxic effects have been directly associated to these molecules [32].

**Table 5.** Known peptidase inhibitors matched to the proteomically identified toxins from *N. mandalayensis* venom.

Description	Accession	10lgP^1^	Peptides^2^	Avg. mass^3^ (Da)	Organism^4^
Kunitz-type serine protease inhibitor	P20229	187	7	6371	*N. naja*
Kunitz-type serine protease inhibitor 2	P00986	117	3	6466	*N. nivea*
Cystatin	P81714	20.33	1	10995	*N. atra*

^1^Peaks Suite confidence parameter. The cutoff was set > 50. ^2^Matched peptides supporting the protein identification. ^3^Retrieved theoretical value. ^4^Species from which the matching toxins were identified.

## Conclusion

The current work comprises the first characterization of the venom proteome of *N. mandalayenses*. The findings presented here will enhance future *Naja* venom comparative studies, as 86 *N. mandalayensis* proteins can now be encompassed in future research from proteomic and/or evolutionary perspectives. The selected approach, i.e., the initial venom fractionation by RP-HPLC, allowed mass spectrometric analyses optimization by removing the large concentration of the dynamic range variation naturally present in the venom, thus allowing the characterization of different toxins with high reliability. There are three major findings in the present study: (1) 3FTx are the major components of the venom; (2) SVMPs are the most diverse group of enzymes found, and; (3) the molecular diversity of the venom is likely to be a direct consequence of the venom spitting evolutionary strategy developed by *N. mandalayensis*, when compared other *Naja* venoms. Regarding the later observation, one can speculate that the most efficiently absorbed molecules (mucosa) would have been selected (as toxins) throughout evolution, such as the cardiotoxins here reported (Figure 2B). The current proteome description should help shed a light on the evolutionary and phylogenetic relations between *N. mandalayenses* and other *Naja*, since the current species, although geographically limited to southeastern Asia and consequently related to the Asian non-spitting cobras, has adopted the spitting strategy present either on the insular southeastern countries or in the African elapids.

## Supplementary material

The following online material is available for this article:

Additional file 1. Other proteins matched to the proteomically identified toxins from *N. mandalayensis* venom.
