# Supplementary material for: Proteomic characterization of Naja mandalayensis venom
Source: J Venom Anim Toxins Incl Trop Dis. 2021 Jul 30;27:e20200125. doi: 10.1590/1678-9199-JVATITD-2020-0125 (PMC8331017; doi:10.1590/1678-9199-JVATITD-2020-0125)
Supplement: Additional file 1. [file 1678-9199-jvatitd-27-e20200125-s1.pdf]

Journal of Venomous Animals and Toxins including Tropical Diseases
https://doi.org/10.1590/1678-9199-JVATITD-2020-0125

Supplementary Material to “Proteomic characterization of *Naja mandalayensis* venom”

Additional file 1. Other proteins matched to the proteomically identified toxins from *N. mandalayensis* venom.

<table>
<tr><th>Description</th><th>Accession</th><th>-10lgP[1]</th><th>Peptides[2]</th><th>Avg. mass[3] (Da)</th><th>Organism[4]</th></tr>
<tr><td>Cobra venom factor</td><td>Q91132</td><td>195.65</td><td>22</td><td>184517</td><td rowspan="2">N. kaouthia</td></tr>
<tr><td>Thaicobrin</td><td>P82885</td><td>140.19</td><td>9</td><td>12038</td></tr>
<tr><td>Complement C3</td><td>Q01833</td><td>167.1</td><td>17</td><td>184926</td><td rowspan="2">N. naja</td></tr>
<tr><td>Cobra serum albumin</td><td>Q91134</td><td>110.33</td><td>10</td><td>69799</td></tr>
<tr><td>Cysteine-rich venom protein kaouthin-2</td><td>P84808</td><td rowspan="2">104.03</td><td>6</td><td>26216</td><td>N. kaouthia</td></tr>
<tr><td>Cysteine-rich venom protein natrin-2</td><td>Q7ZZN8</td><td>6</td><td>26246</td><td rowspan="2">N. atra</td></tr>
<tr><td>Natriuretic peptide Na-NP</td><td>D9IX97</td><td>98.11</td><td>4</td><td>17345</td></tr>
<tr><td>Nerve growth factor beta polypeptide</td><td>B8QCJ8</td><td rowspan="2">44.57</td><td>2</td><td>21713</td><td>N. kaouthia</td></tr>
<tr><td>Venom nerve growth factor 1</td><td>Q5YF90</td><td>2</td><td>27551</td><td>N. sputatrix</td></tr>
<tr><td>Homeobox protein A13</td><td>X2CVW4</td><td>39.11</td><td>1</td><td>24995</td><td>N. atra</td></tr>
<tr><td>Valosin-containing protein p97/p47 complete interacting protein 1</td><td>K4GSG4</td><td>38.1</td><td>2</td><td>26713</td><td rowspan="2">N. kaouthia</td></tr>
<tr><td rowspan="6">Zinc finger E-box binding homeobox 1</td><td>A0A5B9CD30</td><td rowspan="6">33.78</td><td>2</td><td>29144</td></tr>
<tr><td>A0A5B9CDB9</td><td>2</td><td>29144</td><td rowspan="3">N. atra</td></tr>
<tr><td>A0A5B9CDL7</td><td>2</td><td>29144</td></tr>
<tr><td>A0A5B9CCU7</td><td>2</td><td>29144</td></tr>
<tr><td>A0A5B9CCM9</td><td>2</td><td>29144</td><td>N. kaouthia</td></tr>
<tr><td>A0A5B9CCG9</td><td>2</td><td>29144</td><td>N. atra</td></tr>
<tr><td rowspan="3">Titin (Fragment)</td><td>A0A5B9C9W1</td><td rowspan="3">33.77</td><td>2</td><td>30609</td><td rowspan="2">N. kaouthia</td></tr>
<tr><td>A0A5B9C901</td><td>2</td><td>37921</td></tr>
<tr><td>A0A5B9CAH8</td><td>2</td><td>37927</td><td>N. atra</td></tr>
<tr><td>Ubinuclein 1</td><td>A0A3G2KVD1</td><td>31</td><td>1</td><td>14441</td><td>N. melanoleuca</td></tr>
<tr><td rowspan="3">SH3 domain-binding protein 4</td><td>A0A5B9C9Y5</td><td rowspan="3">29.71</td><td>2</td><td>43444</td><td>N. kaouthia</td></tr>
<tr><td>A0A5B9CB97</td><td>2</td><td>43444</td><td rowspan="3">N. atra</td></tr>
<tr><td>A0A5B9CAY5</td><td>2</td><td>43460</td></tr>
<tr><td>Brain-derived neurotrophic factor</td><td>A0A1B3B2U5</td><td>29.33</td><td>2</td><td>24709</td></tr>
</table>

<table>
<tr><th>Description</th><th>Accession</th><th>-10lgP[1]</th><th>Peptides[2]</th><th>Avg. mass[3] (Da)</th><th>Organism[4]</th></tr>
<tr><td>Transient receptor potential cation channel subfamily V member 1</td><td>G9FK64</td><td>28.59</td><td>2</td><td>79797</td><td rowspan="2"></td></tr>
<tr><td>Transient receptor potential cation channel subfamily A member 1</td><td>G9FK44</td><td>28.11</td><td>2</td><td>115457</td></tr>
<tr><td>Myeloid/lymphoid or mixed-lineage leukemia</td><td>K4GUC6</td><td>28.22</td><td>1</td><td>30777</td><td>N. kaouthia</td></tr>
<tr><td rowspan="5">Zinc finger protein FOG family member 2</td><td>A0A5B9CET9</td><td rowspan="5">25.8</td><td>2</td><td>29396</td><td rowspan="2">N. atra</td></tr>
<tr><td>A0A5B9CDX5</td><td>2</td><td>45149</td></tr>
<tr><td>A0A5B9CF35</td><td>2</td><td>45149</td><td>N. kaouthia</td></tr>
<tr><td>A0A5B9CDK0</td><td>2</td><td>45092</td><td>N. atra</td></tr>
<tr><td>A0A5B9CDR5</td><td>2</td><td>45092</td><td rowspan="2">N. kaouthia</td></tr>
<tr><td rowspan="3">Recombination activating protein 1</td><td>G1APK0</td><td rowspan="3">24.53</td><td>1</td><td>38263</td></tr>
<tr><td>G1AP67</td><td>1</td><td>38273</td><td>N. melanoleuca</td></tr>
<tr><td>B2CAW7</td><td>1</td><td>40355</td><td>N. naja</td></tr>
<tr><td rowspan="4">Sacsin molecular chaperone</td><td>A0A5B9CB32</td><td rowspan="5">23.63</td><td>1</td><td>40837</td><td>N. atra</td></tr>
<tr><td>A0A5B9CBL3</td><td>1</td><td>40865</td><td>N. kaouthia</td></tr>
<tr><td>A0A5B9CBP8</td><td>1</td><td>40938</td><td>N. atra</td></tr>
<tr><td>A0A5B9CBY1</td><td>1</td><td>40966</td><td>N. kaouthia</td></tr>
<tr><td>Sacsin</td><td>I6U4Y6</td><td>1</td><td>83143</td><td>N. atra</td></tr>
<tr><td>Solute carrier family 30 member 1</td><td>B9TWF9</td><td>23.57</td><td>1</td><td>19287</td><td rowspan="4">N. kaouthia</td></tr>
<tr><td>Olfactory receptor</td><td>D0VZ37</td><td>23.45</td><td>1</td><td>24152</td></tr>
<tr><td>MutS protein 6</td><td>K4GRF6</td><td>23.4</td><td>1</td><td>25198</td></tr>
<tr><td>Activity-dependent neuroprotector</td><td>K4HH51</td><td>23.08</td><td>1</td><td>17093</td></tr>
<tr><td rowspan="3">Envoplakin</td><td>A0A5B9CHL1</td><td rowspan="3">22.72</td><td>1</td><td>39339</td><td>N. atra</td></tr>
<tr><td>A0A5B9CH67</td><td>1</td><td>39452</td><td>N. kaouthia</td></tr>
<tr><td>A0A5B9CHW8</td><td>1</td><td>39452</td><td>N. atra</td></tr>
<tr><td>Solute carrier family 8 member 1</td><td>K4GS89</td><td>22.43</td><td>1</td><td>33916</td><td>N. kaouthia</td></tr>
<tr><td>Homeobox protein B6</td><td>F5APE6</td><td rowspan="2">22.4</td><td>1</td><td>8389</td><td rowspan="5">N. atra</td></tr>
<tr><td>Homeobox protein A6</td><td>F5APD5</td><td>1</td><td>7294</td></tr>
<tr><td>Homeobox protein A5</td><td>X2CW65</td><td>20.8</td><td>1</td><td>21204</td></tr>
<tr><td rowspan="5">Cytochrome b</td><td>Q2MYZ7</td><td rowspan="5">30.62</td><td>1</td><td>11003</td></tr>
<tr><td>A0A0A7AA57</td><td>1</td><td>11533</td></tr>
<tr><td>Q7YC01</td><td>1</td><td>26950</td><td>N. nigricollis</td></tr>
<tr><td>Q7YBZ7</td><td>1</td><td>26940</td><td rowspan="2">N. pallida</td></tr>
<tr><td>Q7YBZ8</td><td>1</td><td>26974</td></tr>
</table>

Description	Accession	-10lgP[1]	Peptides[2]	Avg. mass[3] (Da)	Organism[4]
	Q7YBZ6		1	26995	
DNA ligase	G3EL08	21.38	1	39776	*N. atra*
	A0A5B9CR38		1	39638	
	A0A5B9CQ27		1	39638	*N. kaouthia*
Leucine rich repeat neuronal 1	K4HJZ7		1	25400	
Periplakin	G3EL90	46.23	5	79620	*N. atra*
	A0A5B9CLD3	30.1	3	55879	*N. kaouthia*
	A0A5B9CN23		3	55879	*N. atra*
KIAA1239	I6U8S8	30.67	1	64498	
Galanin receptor 1	K4GRL2	20.61	1	16134	*N. kaouthia*
Rhodopsin	A0A1B3IJL4	22.35	1	36830	

[1]Peaks Suite confidence parameter. The cutoff was set > 20. [2]Matched peptides supporting the protein identification. [3]Retrieved theoretical value. [4]Species from which the matching toxins were identified.
